# Supplementary material for: Tracking a spin-polarized superconducting bound state across a quantum phase transition
Source: Nat Commun. 2024 Jan 11;15:459. doi: 10.1038/s41467-024-44708-2 (PMC10784290; doi:10.1038/s41467-024-44708-2)
Supplement: Supplementary file 1 — Supplementary Information [file 41467_2024_44708_MOESM1_ESM.pdf]

Supplementary Information for "Tracking a Spin-Polarized Superconducting Bound State across a Quantum Phase Transition"

Sujoy Karan,[1,*] Haonan Huang,[1] Alexander Ivanovic,[2] Ciprian Padurariu,[2]
Björn Kubala,[2,3] Klaus Kern,[1,4] Joachim Ankerhold,[2] and Christian R. Ast[1,†]

[1]*Max Planck Institute for Solid State Research, Heisenbergstraße 1, 70569 Stuttgart, Germany*
[2]*Institute for Complex Quantum Systems and IQST, Universität Ulm, Albert-Einstein-Allee 11, 89069 Ulm, Germany*
[3]*Institute for Quantum Technologies, German Aerospace Center (DLR), Wilhelm-Runge-Straße 10, 89081 Ulm, Germany*
[4]*Institut de Physique, Ecole Polytechnique Fédérale de Lausanne, 1015 Lausanne, Switzerland*

SUPPLEMENTARY NOTE 1

Additional Data Sets

The impurity-superconductor coupling can increase or decrease during tip approach for two different junctions. The atomic force acting in a junction at low transmission, where we operate, is weakly attractive, but not zero [1]. The change in atomic forces due to tip displacement results in atomic relaxation which changes the impurity-substrate coupling by pulling on the impurity [2], thereby reducing its bonding strength to the bulk vanadium. Supplementary Figure 1 shows a data set where impurity-substrate coupling decreases with increasing junction transmission. However, structural relaxations in a junction could also lead to a change in the local density of states, such that the effective impurity-substrate coupling may actually increase upon approaching the tip, as has been observed previously [3, 4]. We note that there are also cases where the impurity is more rigidly bound to the tip and, hence, is not susceptible to the atomic forces acting in the junction. In these cases, the YSR energy does not change within the experimentally accessible junction transmission.

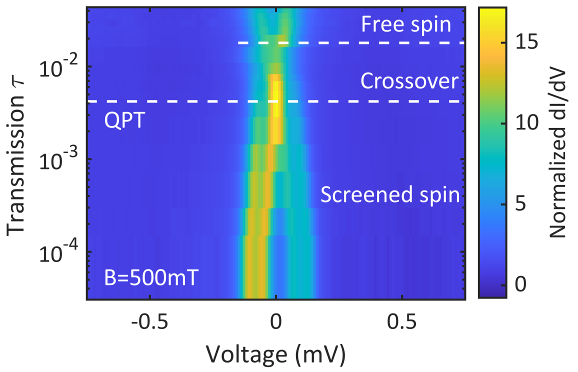

Supplementary Figure 1. Differential conductance map at 500 mT as function of junction transmission. The spin-split YSR state at low transmission indicates that the system initially belongs to the screened spin regime resulting from a stronger impurity-substrate coupling. At high transmission, the system changes its ground state into a doublet (free spin) and no splitting of the state is observed. The splitting observed in the crossover regime occurs because of the thermal excitation.

SUPPLEMENTARY NOTE 2

Theory: Introduction

A single spin-½ impurity gives rise to an in-gap Yu-Shiba-Rusinov state (YSR). Based on the occupation of the YSR state, the system wavefunction can be a spin doublet, when the YSR state is unoccupied, or a singlet, in the opposite case [5–8].

The presence of a magnetic field splits the doublet states. We denote by $|0\uparrow\rangle$ and $|0\downarrow\rangle$ the spin doublet states, that correspond to the free impurity spin. The states indicate that the spin-½ impurity is aligned, respectively anti-aligned, with the external magnetic field. The spin singlet, that corresponds to the screened impurity spin, is denoted by $|1\rangle$. The notation adopted here differs from the notation in the main text, by adding an emphasis on the occupation of the YSR state, that we believe is more intuitive for transport calculations. The notation in the main text for the doublet states $|\uparrow\rangle$ and $|\downarrow\rangle$, indicating the total spin state, is equivalent to the notation here $|0\uparrow\rangle$ and $|0\downarrow\rangle$, indicating an empty YSR state (free impurity spin) and the total spin. The notation in the main text for the singlet state $|0\rangle$, indicating the total spin $S = 0$, is equivalent here to the state $|1\rangle$, indicating the occupation of the YSR state.

The energies of the three states $E_{0\uparrow}$, $E_{0\downarrow}$, and E_1 depend on the exchange coupling strength and give rise to three regimes, as shown in Fig. 3 of the main text. The free spin regime $E_1 > E_{0\uparrow}, E_{0\downarrow}$, a crossover regime $E_{0\uparrow} > E_1 > E_{0\downarrow}$, and the screened spin regime $E_{0\uparrow}, E_{0\downarrow} > E_1$. We have assumed that $E_{0\uparrow} > E_{0\downarrow}$, with the energy difference determined by the Zeeman splitting E_Z of the doublet state $E_{0\uparrow} - E_{0\downarrow} = E_Z$ (as illustrated in Supplementary Fig. 2).

For transport calculations, it is useful to work with fermionic excitation energies. We define the energy required to add a quasiparticle with spin $\uparrow$ to the YSR state as $\epsilon_\uparrow = E_1 - E_{0\downarrow}$. We note that the state involved is $|0\downarrow\rangle$, such that the impurity spin $\downarrow$ is screened by the added quasiparticle $\uparrow$. Similarly, we denote by $\epsilon_\downarrow = E_1 - E_{0\uparrow}$, the energy required to add a quasiparticle with spin $\uparrow$ to the YSR state. Consistent with our assumptions above, we have $\epsilon_\uparrow - \epsilon_\downarrow = E_Z$, therefore $\epsilon_\uparrow > \epsilon_\downarrow$ (as shown in Supplementary Fig. 2).

We note that it is possible to remove either a spin $\uparrow$, or a spin $\downarrow$ quasiparticle from the YSR state, when it is in the singlet state $|1\rangle$. The implication therefore is that transport processes can result in the flip of the impurity

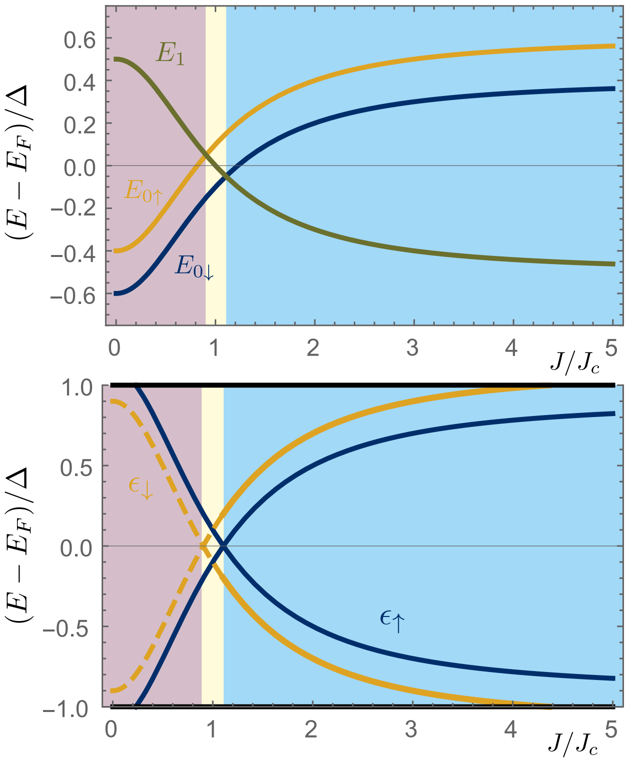

Supplementary Figure 2. Energy of YSR states. Above: energies as a function of exchange coupling. The critical exchange coupling J_c corresponds to the phase transition at zero magnetic field. The Zeeman energy is chosen as $E_Z = 0.1\Delta$. Below: excitation energies as a function of exchange coupling. Both ϵ_σ and $-\epsilon_\sigma$ are shown, for $\sigma = \uparrow, \downarrow$. The dashed lines indicate the excitation energy involves two excited states, while the solid lines indicate excitations from the ground state. The background color indicates the free spin, crossover and screened spin regions, as in Fig. 3 of the main text.

spin, e.g. $|0\downarrow\rangle \xrightarrow{\text{add}\uparrow} |1\rangle \xrightarrow{\text{remove}\downarrow} |0\uparrow\rangle$. Crucially, also the transport between the impurity and its host superconducting substrate can lead to such spin flip, as pointed out in Ref. [9].

Rate equations

The simplest theoretical framework that captures the transport properties of the Zeeman split states takes the form of a rate equation for the probabilities to be in one of three states, $P_{0\uparrow}$, $P_{0\downarrow}$, and P_1. We denote by $\Gamma_{1\uparrow}$ ($\Gamma_{1\downarrow}$) the rate to *remove* a quasiparticle with spin $\uparrow$ ($\downarrow$) from the YSR state, and by $\Gamma_{2\uparrow}$ ($\Gamma_{2\downarrow}$) the rate to *add* a quasiparticle with spin $\uparrow$ ($\downarrow$) to the YSR state.

The rates represent a sum of all the contributing processes, intrinsic processes and tunneling processes, and depend on the bias voltage and the YSR state energy, that we parameterize by the exchange coupling strength J (see Supplementary Fig. 2).

The probabilities characterizing the three states obey the following rate equations,

$$\dot{P}_{0\downarrow} = \Gamma_{1\uparrow}P_1 - \Gamma_{2\uparrow}P_{0\downarrow}, \tag{1}$$
$$\dot{P}_{0\uparrow} = \Gamma_{1\downarrow}P_1 - \Gamma_{2\downarrow}P_{0\uparrow}, \tag{2}$$
$$\dot{P}_1 = \left(\Gamma_{2\uparrow}P_{0\downarrow} + \Gamma_{2\downarrow}P_{0\uparrow}\right) - \left(\Gamma_{1\uparrow} + \Gamma_{1\downarrow}\right)P_1, \tag{3}$$

with the normalization condition $P_{0\uparrow} + P_{0\downarrow} + P_1 = 1$.

The steady state probabilities are given by

$$P_{0\uparrow} = \frac{\Gamma_{1\downarrow}\Gamma_{2\uparrow}}{D}, \quad P_{0\downarrow} = \frac{\Gamma_{1\uparrow}\Gamma_{2\downarrow}}{D}, \quad P_1 = \frac{\Gamma_{2\uparrow}\Gamma_{2\downarrow}}{D}. \tag{4}$$

Where we have used the notation

$$D = \Gamma_{2\uparrow}\Gamma_{2\downarrow} + \Gamma_{1\uparrow}\Gamma_{2\downarrow} + \Gamma_{2\uparrow}\Gamma_{1\downarrow}.$$

In the following, we discuss the rates, which consist of intrinsic rates ($\Gamma_{1\sigma}^{(i)}$, $\Gamma_{2\sigma}^{(i)}$) and tunneling rates ($\Gamma_{1\sigma}^{(t)}$, $\Gamma_{2\sigma}^{(t)}$), with $\sigma \in \{\uparrow, \downarrow\}$, such that

$$\Gamma_{1\sigma} = \Gamma_{1\sigma}^{(i)} + \Gamma_{1\sigma}^{(t)} \quad \text{and} \quad \Gamma_{2\sigma} = \Gamma_{2\sigma}^{(i)} + \Gamma_{2\sigma}^{(t)}.$$

Intrinsic rates

The occupation of the YSR state can change due to intrinsic processes involving the quasiparticle population above the superconducting gap. We stress that these processes do not involve tunneling between the tip and substrate. The intrinsic process that describes the addition of a quasiparticle to the YSR state, changing the state from $|0\sigma\rangle$, with $\sigma \in \{\uparrow, \downarrow\}$, to the singlet state $|1\rangle$, requires that the quasiparticle has spin $\bar{\sigma}$, opposite to σ. We denote the corresponding rate by $\Gamma_{2\bar{\sigma}}^{(i)}$. Similarly, the rate to emit a quasiparticle with spin $\bar{\sigma}$ into the continuum, is denoted by $\Gamma_{1\bar{\sigma}}^{(i)}$. The latter process transforms the initial state $|1\rangle$ into the doublet state $|0\sigma\rangle$.

In absence of tunneling between tip and substrate, the intrinsic processes in the tip and are responsible for the equilibrium value of the YSR state occupation. We apply detailed balance to determine the relations between the intrinsic rates. The equilibrium population in each state in absence of tunneling $P_{0\uparrow}^{(\mathrm{eq})}$, $P_{0\downarrow}^{(\mathrm{eq})}$, and $P_1^{(\mathrm{eq})}$, are obtained from Eqs. 4 by setting the tunneling rates to zero, such that $\Gamma_{1\sigma} = \Gamma_{1\sigma}^{(i)}$ and $\Gamma_{2\sigma} = \Gamma_{2\sigma}^{(i)}$. We require that the equilibrium populations are related by the Fermi-Dirac distribution

$$\frac{P_1^{(\mathrm{eq})}}{P_{0\downarrow}^{(\mathrm{eq})}} = \frac{\Gamma_{2\uparrow}^{(i)}}{\Gamma_{1\uparrow}^{(i)}} = \frac{n_\mathrm{F}(\epsilon_\uparrow)}{1 - n_\mathrm{F}(\epsilon_\uparrow)}, \qquad \frac{P_1^{(\mathrm{eq})}}{P_{0\uparrow}^{(\mathrm{eq})}} = \frac{\Gamma_{2\downarrow}^{(i)}}{\Gamma_{1\downarrow}^{(i)}} = \frac{n_\mathrm{F}(\epsilon_\downarrow)}{1 - n_\mathrm{F}(\epsilon_\downarrow)}.$$

Furthermore, we will assume that the rate to emit a quasiparticle with spin σ into the continuum, transition from $|0\bar{\sigma}\rangle$ to state $|1\rangle$, is independent of the orientation of the impurity spin $\bar{\sigma}$. Therefore, we have

$$\Gamma_{1\uparrow}^{(i)} = \Gamma_{1\downarrow}^{(i)} = \Gamma_1^{(i)}.$$

This assumption is not necessary, but convenient to reduce the number of parameters of the model. The physical mechanism behind such intrinsic processes, as well as

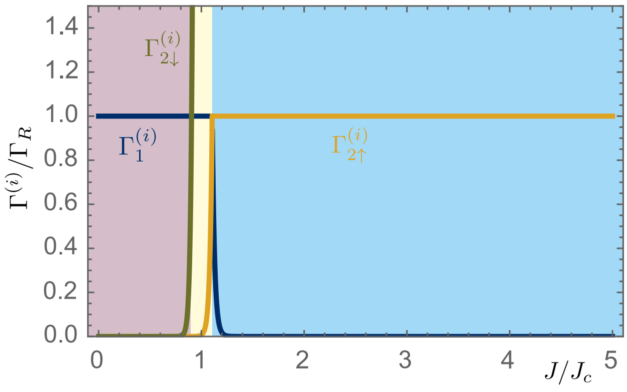

Supplementary Figure 3. Intrinsic rates as a function of exchange coupling. The critical exchange coupling J_c corresponds to the phase transition at zero magnetic field.

the origin of the quasiparticle population above the gap at mK temperatures, remain unknown.

The two relations obtained by applying detailed balance, together with our assumption, express the intrinsic rates in terms of a single free parameter, which we denote ΓR, an intrinsic rate of relaxation. We parameterize the intrinsic rates in terms of Γ_R, as follows

$$\Gamma_1^{(i)} = \begin{cases} \Gamma_R, & \epsilon_\uparrow \geq 0, \\ \Gamma_R \exp(\epsilon_\uparrow / k_B T). & \epsilon_\uparrow < 0 \end{cases} \tag{5}$$

$$\Gamma_{2\uparrow}^{(i)} = \begin{cases} \Gamma_R \exp(-\epsilon_\uparrow / k_B T), & \epsilon_\uparrow \geq 0, \\ \Gamma_R, & \epsilon_\uparrow < 0. \end{cases} \tag{6}$$

$$\Gamma_{2\downarrow}^{(i)} = \begin{cases} \Gamma_R \exp(-\epsilon_\downarrow / k_B T), & \epsilon_\uparrow \geq 0, \\ \Gamma_R \exp\left[(\epsilon_\uparrow - \epsilon_\downarrow)/k_B T)\right], & \epsilon_\uparrow < 0. \end{cases} \tag{7}$$

The intrinsic rates are shown in Supplementary Fig. 3. The parametrization chose ensures that the intrinsic rates $\Gamma_1^{(i)}$ and $\Gamma_{2\uparrow}^{(i)}$ are bound by Γ_R. The intrinsic rate $\Gamma_{2\downarrow}^{(i)}$ becomes much larger than Γ_R in the regime $\epsilon_\uparrow < 0$, indicating that the higher excited state $|0\uparrow\rangle$ relaxes to the ground state $|1\rangle$ in this regime at a rate much faster than the relaxation of the lower excited state $|0\downarrow\rangle$.

Tunneling rates

We assume that the tunneling process is spin-conserving and characterized by a spin- and momentum-independent tunneling amplitude t. The density of states of the substrate is denoted $\rho_\sigma(\omega)$, that may differ for the two spin species $\sigma = \{\uparrow, \downarrow\}$. The chemical potentials for different spin species align and are denoted by μ_s for the substrate and μ_t for the tip, respectively. The occupation of the electronic states of the substrate is given by the Fermi-Dirac distribution $n_F(\omega)$.

In the experiment, the magnetic field is sufficiently large such that the substrate is in the normal-conducting state. This provides a simplification, since the density of states is approximately flat at the scale of the Zeeman splitting E_Z, and therefore becomes spin-independent.

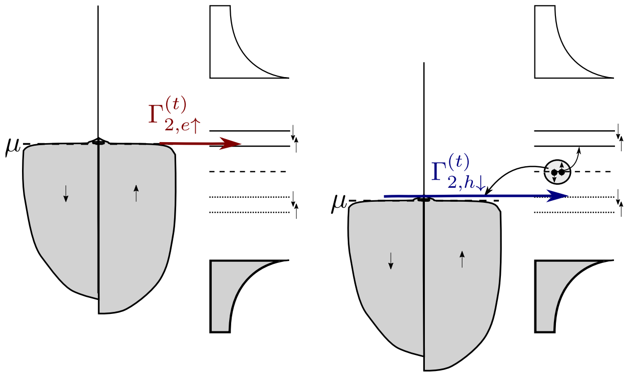

Supplementary Figure 4. Illustration of two transport processes that add a spin $\uparrow$ quasiparticle to the YSR state, one realized by transporting a spin $\uparrow$ electron, the other realized by transporting a spin $\downarrow$ hole. The latter is equivalent to an electron with spin $\downarrow$ resulting from a Cooper pair splitting event, traveling in the opposite direction of the hole.

However, we will provide expressions for the rate equations that can account for a future experimental situation where the substrate density of states could be potentially spin-dependent.

The rates describing tunneling processes contribute to the total rates $\Gamma_{1\sigma}$ and $\Gamma_{2\sigma}$, as follows. When the initial state is either one of the doublets, $|0\sigma\rangle$, a tunneling process will add a quasiparticle with spin $\bar{\sigma}$, opposite σ, resulting in the singlet. We distinguish two possibilities: either i. an electron with spin $\bar{\sigma}$ tunnels into the YSR state, with rate denoted by $\Gamma_{2,e\bar{\sigma}}^{(t)}$; or ii. a hole with spin σ tunnels into the YSR state, with rate denoted by $\Gamma_{2,h\sigma}^{(t)}$ (see also Supplementary Fig. 4). Since both processes create a quasiparticle excitation with spin $\bar{\sigma}$ in the YSR state, they add up to give the contribution to $\Gamma_{2\bar{\sigma}}$ due to tunneling, denoted $\Gamma_{2\bar{\sigma}}^{(t)}$.

$$\begin{aligned} \Gamma_{2\bar{\sigma}}^{(t)} &= \Gamma_{2,e\bar{\sigma}}^{(t)} + \Gamma_{2,h\sigma}^{(t)}. \\ \Gamma_{2,e\bar{\sigma}}^{(t)} &= 2\pi |u|^2 |t|^2 \rho_{\bar{\sigma}}(eV + \epsilon_{\bar{\sigma}}) n_F(eV + \epsilon_{\bar{\sigma}}). \\ \Gamma_{2,h\sigma}^{(t)} &= 2\pi |v|^2 |t|^2 \rho_\sigma(eV - \epsilon_{\bar{\sigma}}) \bar{n}_F(eV - \epsilon_{\bar{\sigma}}). \end{aligned}$$

In the expressions above, we have introduced the coherence factors of the YSR state, u and v. We denoted by $|u|^2$ the probability to add an electron to the YSR state, while $|v|^2$ represents the probability to add a hole, respectively. Furthermore, we have used the convention that $eV = \mu_t - \mu_s$, where μ_s and μ_t are the chemical potentials of the substrate and tip, respectively. We have also introduced the common notation $\bar{n}_F(\omega) = 1 - n_F(\omega)$ to denote the occupation for holes.

Similarly, we obtain the rates of tunneling processes that remove a quasiparticle from the YSR state, leading to the transition from state $|1\rangle$ to one of the dublet states $|0\sigma\rangle$. A quasiparticle can be removed either by removing from the YSR state an electron with spin $\bar{\sigma}$, $\Gamma_{1,e\bar{\sigma}}^{(t)}$, or a hole with spin σ, $\Gamma_{1,h\sigma}^{(t)}$. We find in analogy to the results for

adding a quasiparticle above,

$$\begin{aligned}\varGamma^{(t)}_{1\bar\sigma} =&\varGamma^{(t)}_{1,e\bar\sigma} + \varGamma^{(t)}_{1,h\sigma}.\\ \varGamma^{(t)}_{1,e\bar\sigma} =&2\pi|u|^2|t|^2\rho_{\bar\sigma}(eV+\epsilon_{\bar\sigma})\bar n_{\mathrm{F}}(eV+\epsilon_{\bar\sigma}).\\ \varGamma^{(t)}_{1,h\sigma} =&2\pi|v|^2|t|^2\rho_{\sigma}(eV-\epsilon_{\bar\sigma})n_{\mathrm{F}}(eV-\epsilon_{\bar\sigma}).\end{aligned}$$

Steady state current

The electrical current is expressed in terms of tunneling rates and the steady state probabilities. The latter are given by Eq. (4), with the total rates $\varGamma_{1\sigma} = \varGamma^{(i)}_1 + \varGamma^{(t)}_{1\sigma}$ and $\varGamma_{2\sigma} = \varGamma^{(i)}_{2\sigma} + \varGamma^{(t)}_{2\sigma}$, given by sum of intrinsic and tunneling rates.

The total steady state current is given by the expression,

$$I = e\left[\sum_\sigma \left(\varGamma^{(t)}_{2,e\bar\sigma} - \varGamma^{(t)}_{2,h\sigma}\right)P_{0\sigma} + \left(\varGamma^{(t)}_{1,h\sigma} - \varGamma^{(t)}_{1,e\bar\sigma}\right)P_1\right]. \quad (8)$$

The expression accounts for all charge transfer processes across the tip-substrate junction. The first term accounts for the possibility to add a quasiparticle to the YSR state $|0\sigma\rangle$ either by transporting an electron with spin $\bar\sigma$, or a hole with spin σ. The second term, similarly, accounts for the possibility to remove a quasiparticle with spin $\sigma =\uparrow$ or $\downarrow$ from the YSR state $|1\rangle$, by transporting an electron with spin $\bar\sigma$, or a hole with spin σ.

The total current can be further understood in terms of elementary transport processes. An elementary transport process consists of two transitions: the first changes the occupation of the YSR state, and the second restores the original occupation, thereby completing the transport cycle. We distinguish two types of elementary transport processes: i. when one transition occurs due to a tunneling process, while the other transition is intrinsic, such that a total charge e is transported; and ii. when both transitions occur due to a tunneling process, such that a total charge $2e$ is transported by sequential charge e tunneling events. Supplementary Fig. 5 illustrates an example of the two types of processes.

The total steady state current is the sum of currents contributed by the two types of processes,

$$I = I_e + I_{2e}.$$

Charge e elementary transport process

For this transport process, charge is transported in a single tunneling event. We must account both for forward transport and for backward transport, as follows.

$$I_e = e\sum_{\sigma\sigma'}\frac{\left(\varGamma^{(t)}_{2,e\bar\sigma} - \varGamma^{(t)}_{2,h\sigma}\right)\varGamma^{(i)}_1 + \varGamma^{(i)}_{2\bar\sigma}\left(\varGamma^{(t)}_{1,h\sigma'} - \varGamma^{(t)}_{1,e\bar\sigma'}\right)}{\left(\varGamma_{1\uparrow}+\varGamma_{1\downarrow}\right)}P_{0\sigma}. \quad (9)$$

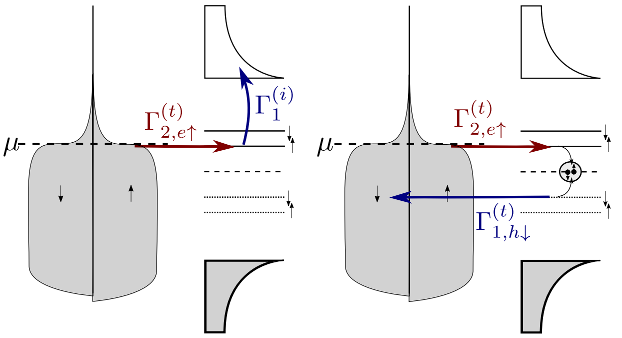

Supplementary Figure 5. Two types of elementary transport processes. Left: illustration of an elementary transport process carrying charge e. In the first step, a tunneling event leads to the transition $|0\downarrow\rangle$ to $|1\rangle$. In a second step, the state $|1\rangle$ relaxes back to state $|0\downarrow\rangle$ by an intrinsic process without charge transport. Right: illustration of an elementary transport process carrying charge $2e$. The first step is identical to the left illustration. In the second step, a hole tunnels out of the YSR state, restoring the state $|0\downarrow\rangle$ and leading to a total $2e$ charge transfer. The hole tunneling can be seen as an electron tunneling in the opposite direction, forming a Cooper pair with the electron that tunneled in the first step.

The first term, proportional to $P_{0\sigma}$, describes the process when an electron, or a hole, tunnels into the YSR state changing the occupation from $|0\sigma\rangle$ to $|1\rangle$, as shown in Supplementary Fig. 4. The second step of the elementary process occurs therefore via an intrinsic process, restoring an unoccupied YSR state $|0\sigma'\rangle$, without charge transport. The total process is depicted in the left side of Supplementary Fig. 5. Alternatively, the transition from $|0\sigma\rangle$ to $|1\rangle$ could occur by an intrinsic process, while the second step, from $|1\rangle$ to $|0\sigma'\rangle$, could occur by tunneling.

Note that the information about the energy of the states and filling factors of the substrate are all encoded in the rates and indirectly, in the steady state probabilities. Therefore, the expressions apply for all regimes, free spin, intermediate, as well as the screened spin regime.

Charge 2e elementary transport process

When both steps of the elementary transport process involve a tunneling event, such as the process depicted in the right side of Supplementary Fig. 5, the total transported charge is $2e$. The transport of $2e$ charge is reminiscent of Andreev reflection. Indeed, the two charged particles involved in transport change the number of Cooper pairs in the tip condensate by one. However, there is also an important difference, the process described here is a sequential process consisting of two single particle transport events.

Elementary transport processes involving two sequential tunneling events have the following structure,

$$|0\sigma\rangle \xrightarrow{\varGamma^{(t)}_{2,e\bar\sigma};\varGamma^{(t)}_{2,h\sigma}} |1\rangle \xrightarrow{\varGamma^{(t)}_{1,h\sigma'};\varGamma^{(t)}_{1,e\bar\sigma'}} |0\sigma'\rangle\,.$$

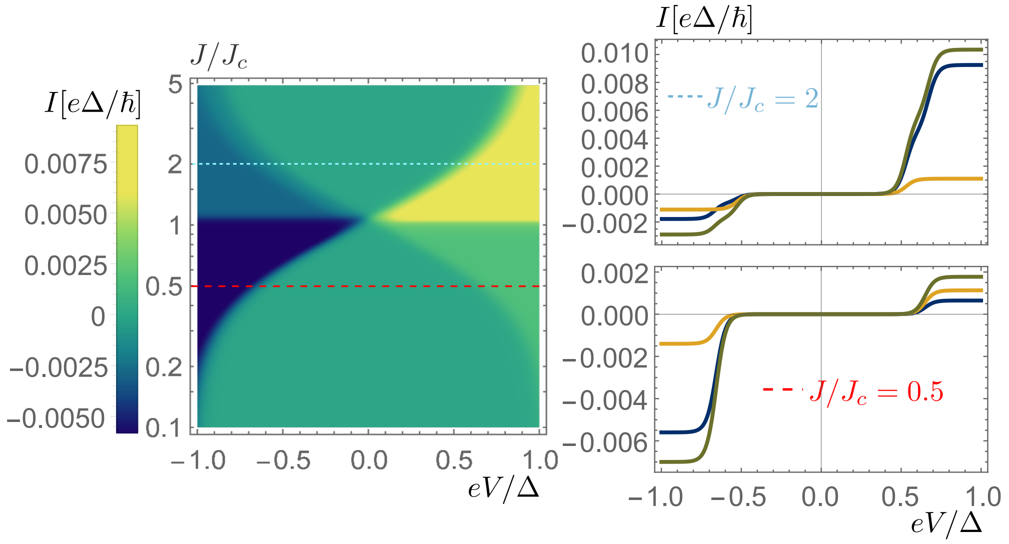

Supplementary Figure 6. Current-voltage curves. Left: Density plot of the current as a function of voltage V and exchange coupling J. Right: cuts showing the I-V curves before and after the phase transition. The blue and gold curves show the contributions I_e and I_{2e}, respectively, while the green curves show the total current.

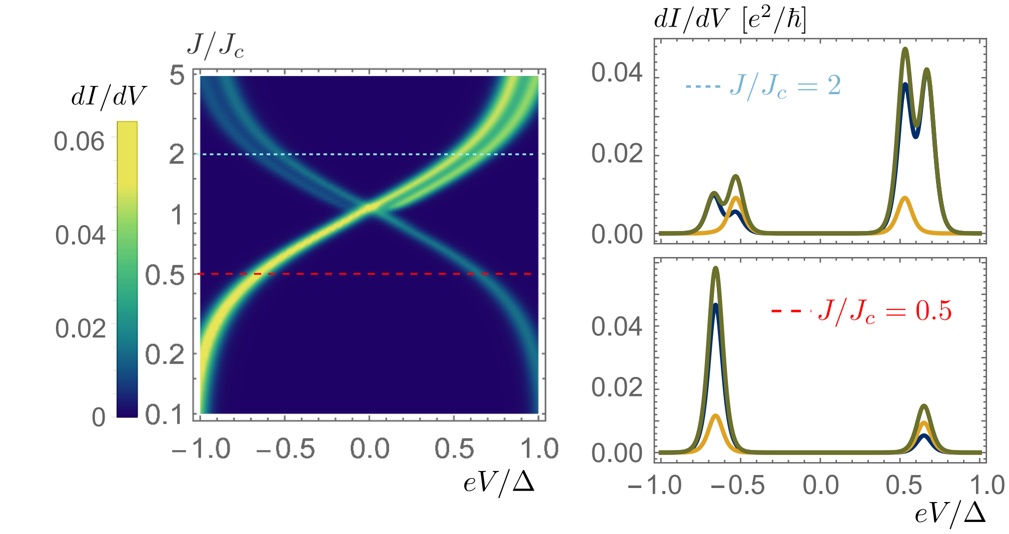

Supplementary Figure 7. Differential conductance curves. Left: Density plot of the differential conductance as a function of voltage V and exchange coupling J. Right: cuts showing the differential conductance curves before and after the phase transition. The blue and gold curves show the contributions dI_e/dV and dI_{2e}/dV, respectively, while the green curves show the differential conductance.

The combination of $\Gamma^{(t)}_{2,e\bar{\sigma}}$ and $\Gamma^{(t)}_{1,e\bar{\sigma}'}$ describes an electron tunneling back and forth across the junction, giving rise to current noise, but without net charge transport. Similarly, the combination of $\Gamma^{(t)}_{2,h\sigma}$ and $\Gamma^{(t)}_{1,h\sigma'}$ describes tunneling back and forth of a hole.

The transport of $2e$ charge arises from combining tunneling of an electron into the tip $\Gamma^{(t)}_{2,e\bar{\sigma}}$ with the subsequent tunneling of a hole out of the tip $\Gamma^{(t)}_{1,h\sigma'}$. Note that, as a consequence of the assumption in our model that tunneling can flip the spin of the impurity, all combinations $\{\sigma, \sigma'\}$ are possible for the spin of the tunneling electron and hole.

While the process above transports a charge $2e$ from the substrate to the tip, the reverse process is obtained by combining $\Gamma^{(t)}_{2,h\sigma}$ with $\Gamma^{(t)}_{1,e\bar{\sigma}'}$, which transports a charge $2e$ from the tip to the substrate.

The total resulting current is given by the sum over all possible $2e$ processes

$$I_{2e} = 2e \sum_{\sigma\sigma'} \left(\frac{\Gamma^{(t)}_{1,h\sigma'} \Gamma^{(t)}_{2,e\bar{\sigma}}}{(\Gamma_{1\uparrow} + \Gamma_{1\downarrow})} - \frac{\Gamma^{(t)}_{2,h\sigma} \Gamma^{(t)}_{1,e\bar{\sigma}'}}{(\Gamma_{1\uparrow} + \Gamma_{1\downarrow})} \right) P_{0\sigma}. \qquad (10)$$

We note that typically, only a few of the terms contribute significantly to the total current.

Results

The experimentally relevant regime is defined by $\Delta \simeq 0.6\ meV$, $E_Z \simeq 0.07\Delta$ corresponding to $B = 750$ mT and a g-factor of $g = 2$, and $k_B T \simeq 0.014\Delta$. In this regime, the results reproduce the steps in current that arise when the voltage aligns the chemical potential of the substrate with an electronic transition of the tip YSR state (see Supplementary Fig. 6). These current steps manifest as peaks in the differential conductance, as seen in Supplementary Fig. 7.

In the free spin regime, a single peak is found in the differential conductance, corresponding to the electronic transition from the ground state $|0\downarrow\rangle$ to the singlet state $|1\rangle$. Transport processes starting in the higher energy spin state $|0\uparrow\rangle$ are thermally suppressed. In contrast, in the screened spin regime, two peaks are seen, contributed by transitions from the ground state $|1\rangle$ to either excited states $|0\downarrow\rangle$ or $|0\uparrow\rangle$. The distance between the two peaks

is directly given by twice the Zeeman energy.

Both charge e and charge $2e$ elementary processes contribute significantly in the experimentally relevant parameter regime, although typically charge e processes dominate.

The theoretical result presented in Fig. 3 of the main text, accounts for the fact that in the experiment, approaching the tip increases the transmission of the tunnel barrier, $\tau \equiv |t|^2$, and also modifies the effective exchange coupling J, that leads to the modification of the YSR state excitation energies, $\epsilon_\uparrow$ and $\epsilon_\downarrow$. We have used the simplest method to account for the simultaneous dependencies, by assuming a linear dependence of the YSR excitation energies on the transmission τ,

$$\epsilon_\sigma(\tau) = \alpha\tau + \epsilon_0 + \sigma E_Z/2. \tag{11}$$

With this linear dependence, the result of the rate equation model reproduces well the measured data. The parameters α and ϵ_0 can be fitted independently, using the position of YSR peaks in absence of magnetic field. The only free parameters that concern electronic transport in presence of magnetic field are related to the intrinsic relaxation rate $\Gamma_R/\Delta = 0.01$, the tunneling rate $\Gamma_t/\Delta = 2\pi|t|^2\rho/\Delta = 0.01$, and the asymmetry in the coherence factors of the YSR state, $|u|^2/|v|^2 = 7$. These are fitting parameters for the plot in Fig. 3 in the main text.

SUPPLEMENTARY NOTE 3

Thermometry

As an outlook, we indicate a potential application of the functionalized STM tip to realize thermometry of the quasiparticle temperature at the atomic scale.

At 15 mK, it is not possible to measure the temperature broadening directly from the width of the YSR conductance peak. This is due to fluctuations in the electromagnetic environment that induce a much larger broadening, limiting the energy resolution. We offer a promising workaround based on the current setup, that exploits the tunability of the YSR state on the tip through the phase transition. At the phase transition the conductance shows a pronounced peak centered around zero voltage, as can be seen in Fig. 2(b) and (c) of the main text. The width of this peak in voltage is broad, consistent with environmental fluctuations. However, after integrating the conductance peak across the voltage, we obtain a peak with a well defined width as a function of transmission, the y-axis in Fig. 2(b) and (c). This width can be converted into an energy width using the YSR energy-transimission curve (e.g. the curve in Fig. 2(d)). The resulting width may be significantly narrower than the energy resolution. Our insight, according to the theoretical model presented here, is that this width in units of energy reveals the quasiparticle temperature of the combined tip and sample continuum, as seen by the STM tip.

The theoretical framework sketched above requires experimental verification. The feasibility of the STM as a quasiparticle thermometer cannot be established without a temperature-sensitive study of the transport properties. This substantial effort is outside the scope of the current manuscript.

SUPPLEMENTARY REFERENCES

* s.karan@fkf.mpg.de
† c.ast@fkf.mpg.de

[1] M. Ternes, C. González, C. P. Lutz, P. Hapala, F. J. Giessibl, P. Jelínek, and A. J. Heinrich, *Interplay of conductance, force, and structural change in metallic point contacts,* Physical Review Letters **106**, 016802 (2011).

[2] L. Farinacci, G. Ahmadi, G. Reecht, M. Ruby, N. Bogdanoff, O. Peters, B. W. Heinrich, F. von Oppen, and K. J. Franke, *Tuning the Coupling of an Individual Magnetic Impurity to a Superconductor: Quantum Phase Transition and Transport,* Physical Review Letters **121**, 196803 (2018).

[3] L. Malavolti, M. Briganti, M. Hänze, G. Serrano, I. Cimatti, G. McMurtrie, E. Otero, P. Ohresser, F. Totti, M. Mannini, R. Sessoli, and S. Loth, *Tunable Spin–Superconductor Coupling of Spin 1/2 Vanadyl Phthalocyanine Molecules,* Nano Letters **18**, 7955 (2018).

[4] H. Huang, R. Drost, J. Senkpiel, C. Padurariu, B. Kubala, A. L. Yeyati, J. C. Cuevas, J. Ankerhold, K. Kern, and C. R. Ast, *Quantum phase transitions and the role of impurity-substrate hybridization in Yu-Shiba-Rusinov states,* Communications Physics **3**, 1 (2020).

[5] P. Anderson, *Theory of dirty superconductors,* Journal of Physics and Chemistry of Solids **11**, 26 (1959).

[6] R. Žitko, O. Bodensiek, and T. Pruschke, *Effects of magnetic anisotropy on the subgap excitations induced by quantum impurities in a superconducting host,* Phys. Rev. B **83**, 054512 (2011).

[7] H. Huang, R. Drost, J. Senkpiel, C. Padurariu, B. Kubala, A. L. Yeyati, J. C. Cuevas, J. Ankerhold, K. Kern, and C. R. Ast, *Quantum phase transitions and the role of impurity-substrate hybridization in Yu-Shiba-Rusinov states,* Communications Physics **3**, 199 (2020).

[8] T. Machida, Y. Nagai, and T. Hanaguri, *Zeeman effects on Yu-Shiba-Rusinov states,* Phys. Rev. Res. **4**, 033182 (2022).

[9] W.-V. van Gerven Oei, D. Tanasković, and R. Žitko, *Magnetic impurities in spin-split superconductors,* Phys. Rev. B **95**, 085115 (2017).
